# Whose Knowledge Is Under the Lens? A Contribution to the Debate Sparked by Clark and Walker's 2011 *Research Ethics in Victimization Studies: Widening the Lens*

**DOI:** 10.1177/10778012211038972

**Published:** 2021-11-19

**Authors:** Shiva Nourpanah, Myrna Dawson

**Affiliations:** 1 3690 Saint Mary’s University, Halifax, NS, Canada; 2 University of Guelph, Guelph, ON, Canada

**Keywords:** violence against women, advocacy, victimization research

## Abstract

The starting point for this commentary is the fruitful exchange of ideas on the ethics of victimization research, published in this journal in 2011, sparked by Clark and Walker's article, “Research Ethics in Victimization Studies: Widening the Lens”. This article provoked a flurry of responses that, taken altogether, provide an illuminating cornerstone for the ethical debates and issues surrounding victimization research. It further inspired us to reflect upon and share our experiences on conducting victimization research at that particular intersection of academia and advocacy that we both occupy. What struck us about this exchange was the absence of any discussion about the role of anti-violence against women advocates, service providers, and organizations in victimization research.

## Introduction

The starting point for this commentary is the fruitful exchange of ideas on the ethics of victimization research, published in this journal in 2011, sparked by Clark and Walker's article “Research Ethics in Victimization Studies: Widening the Lens” (2011). This article, critiquing contemporary victimization research as conducted in the business-like and neoliberalized university environment and calling for a deeper integration of ethics in every aspect of research, provoked a flurry of responses. These included Cerulli (2011), Conte (2011), Mulla and Hlavka (2011), and culminated in Walker and Clark's response (2011). Taken altogether, this exchange provides an illuminating cornerstone for the ethical debates and issues surrounding victimization research. It is a must-read for both emerging scholars who are embarking on victimization research and also well-established and published scholars in the field, as exemplified by the first and second authors of this note, Shiva and Myrna.

It further inspired us to reflect upon and share our experiences on conducting victimization research at that particular intersection of academia and advocacy that we both occupy. What struck us about this exchange, as lively, in-depth, rigorous, and multifaceted as it is, was the absence of any discussion about the role of antiviolence against women (VAW) advocates, service providers, and organizations, beyond requesting practical assistance from them. Such assistance could include recruitment of research participants and then subsequent support and care for participants should they become distressed from participating in interviews (see, e.g., Ellsberg & Heise, 2005).

That this sector is largely run by and for women may be partly the explanation for the absence of a role other than that of care and support, as discussed further below. Notwithstanding the fact that there is often overlap between survivors and antiviolence women frontline workers, the idea that women who work with women could provide insight and knowledge about their experiences of violence and the consequences they endure in the aftermath remains minimal in the literature. As an example, we considered the World Health Organization's comprehensive practical guide on how to do research with women who are victims of gender-based violence (Ellsberg & Heise, 2005), an otherwise exemplary piece of gray literature providing abundant, systematic advice on research that may have serious repercussions for the safety and welfare of not only the participants but also the researchers. It was disappointing from our review that the role of community organizations in the women's sector only merited a mention that it may be a “good idea” to invite them into training sessions for interviewers (Ellsberg & Heise, 2005, p. 199).

This minimal approach to the VAW sector led to our further reflection on women's advocacy and its representation and significance in academia. Who is “allowed” to generate knowledge? Whose voices are heard and considered “expert”? It is an interesting question as funding agencies often, and with increasing frequency, require or recommend partnerships with community organizations in academic proposals for research grants—something which has been noted somewhat wryly by both academics, advocates, and/or VAW organizations, many of which operate with explicit feminist philosophies and mandates. Indeed, some latter organizations have recently developed their own guide laying out best principles and practices on how and when to collaborate with academia, urging careful consideration of proposed projects and an analysis of the actual benefits of such projects for their organization (Sexual Violence Research Initiative, 2020). This guide also emphasizes the existing “hierarchy of knowledge” which nullifies some voices while amplifying others and warns of unethical collaborations based on unequal relations of power in which practitioners may be perceived as being in an inferior position vis-a-vis academic researchers (SVRI, 2020). Despite such concerns, there continues to be an effort to reach out and include voices from community and civil society in research design and implementation, for example, “key informant” interviews and the provision of letters of support. But the absence of detailed and rigorous inclusion of these voices as part of the process of generating knowledge remains noteworthy and problematic, as discussed here.

We further note the role of civil society as manifested in community organizations conducting frontline work with marginalized and vulnerable people has been granted significance by academics and government in other arenas, notably in refugee studies, where civil society operations have been strongly theorized (Pries, 2019). As regards the anti-VAW sector, there have been noteworthy attempts to include and theorize the role of practitioners and advocates and their relationship with research dating from the late 1990s, as synthesized by Sullivan et al. (2017) in their discussion of researcher and practitioner collaborations in the criminal justice system. Their review of this literature, which they acknowledge is a “limited scholarship” (p. 890) from almost 30 years ago, reveals the extant shortcomings of such collaborations. Specifically, such collaborative research falls short of systematic inquiries and remains confined largely to case studies and anecdotes, the outcomes of which are not studied. Furthermore, the research is mostly confined to the process of collaboration itself, which is necessary but not sufficient. Sullivan et al.'s (2017) own research with practitioners in the criminal justice system nevertheless demonstrates the potential for positive relationship building:

Reaching out allows researchers and practitioners to build trust and respect, gain a new perspective on partnerships, and ultimately build a more positive view of researchers and their intent, and of practitioners and their assets. (p. 894)

At this point, it is necessary to emphasize that both authors, Shiva and Myrna, see themselves socially positioned not just as academics, but also as strong advocates for the cause of the elimination of violence against women. Shiva, in addition to holding a postdoctoral fellowship at the University of Guelph at the time of this writing, also works in a community organization, the Transition House Association of Nova Scotia, an umbrella association of VAW organizations across the province of Nova Scotia, Canada. In this position, she provides social advocacy for their cause, including mainstream media appearances, both televised and written, and maintains an active social media presence. Likewise, Myrna combines a long, productive academic career with high-profile advocacy, specifically drawing public attention to the ongoing issue of femicide or the killing of women because they are women (Dawson & Carrigan, 2020), most recently, by establishing the Canadian Femicide Observatory for Justice and Accountability, which documents and analyzes cases of women-killing across Canada, and disseminates this information publicly and widely through active social media presence. In this context, the absence of voices from the women's sector has already raised her concern. For example, in a recent publication discussing the operations of Domestic Violence Death Review Panels across six high-income countries, she notes:

An ongoing criticism of many DVDRs is that there is little to no representation from a core group of experts who arguably have the greatest wealth of knowledge about domestic violence—-feminist and/or frontline women's advocates. In most jurisdictions, feminist and grassroots violence against women agencies were largely responsible for lobbying for the establishment of these initiatives. *It is perplexing, then, their voices are not represented to a greater degree*. (Dawson, 2021, p. 683, italics added by the authors)

As such, over the course of their work together through 2019–2021, both authors had ample reason to reflect on the often tense relationship between academia and advocacy, specifically on the representations (or lack thereof) of women's work and advocacy in victimization research. In what follows, after a brief theoretical note on the systemic (de)valuation of women's work generally and the consequences of this, we will provide a critical review of the select literature on victimization research. Although not an exhaustive review, we feel there are significant gaps and, therefore, we call for more meaningful, authentic, and sustained partnerships between academia and advocacy, in this instance, by fostering the relationship between researchers on intimate partner and domestic violence and the anti-VAW organizations mandated to serve and care for the subjects and participants of their research.

## Conceptual Framework

Feminist scholars have discussed the devaluation of “social reproduction,” or what is commonly known as “women's work,” at length (Bakker, 2007; Hartsock, 2006; Mackintosh, 1988; Tastsoglou & Nourpanah, 2022). The global spread of industrial capitalism exacerbated patriarchal gendered divisions of labor, relegating work performed for the maintenance of the “domestic” sphere and tasks associated with care to women. The withdrawal of state funding from services such as health care and education occurring from the 1970s onward further exacerbated the burden of care shouldered by women (Visvanathan et al., 2011). Care work, feminized in the sense that it has been considered the natural duty of women to care for vulnerable members of society, whether children, the sick, the disabled, the elderly, or, in the case of this commentary, victimized women who have experienced violence, has routinely been devalued, defunded, underappreciated, and overlooked, albeit in racialized and historically shifting patterns (Baines et al., 1998; Duffy, 2007). However, we contend that work being done by women to protect other women from violence is not only devalued materially, but also neglected and devalued theoretically. There are varying types of expertise in domestic violence prevention, but a continued hierarchy in which type of expertise is valued and which type is not persisted. This hierarchy reflects the historical and contemporary social structures that have continued to see some types of work—and largely, women's work—as undervalued.

Alongside the feminist understanding of the devaluation of women's work, our conceptual framework draws on feminist research on violence against women as a public, social issue rather than private, criminalized incidents. Under this framework, domestic violence, one of the most prevalent and common forms of violence against women, is seen as connected to and influenced by community and social structures (Fairbairn & Dawson, 2013). Once we consider violence against women in this light, rooted in long-standing patriarchal structures, our view on those who work with victims of such violence may change accordingly. Rather than “just” providing immediate care and succor to women suffering from violence from purely altruistic and charitable impulses, we contribute to a reconsideration of the role of frontline advocates as civil society agents pushing for long-term social change, who hold valuable knowledge in the struggle to eliminate violence against women. This view of advocates and workers in the women's sector is validated when we consider their changing societal role. These grassroots, community-based organizations were initially established to respond to immediate needs in local communities, such as offering a temporary safe space for women and their families escaping unsafe homes. This role has evolved to a great extent, and these organizations find themselves filling much larger gaps in communities where services to vulnerable people have been systematically defunded. Feminist scholars have noted the contributions of activists and advocates in this sector to pushing domestic violence from the private sphere onto the sociopolitical agenda (Sev’er, 2001).

What does the “women's sector” have to contribute to our knowledge and understanding of violence against women? As Myrna argues, women's advocates and frontline workers might be in a position to articulate the intersecting oppressions in women's lives more clearly and effectively than, for example, criminal justice and other government agencies, given their firsthand witnessing of the impacts of such oppressions. They may further develop deeper insight into the role that class, race, and other social identities play in structuring the harms faced by women (Dawson & Carrigan, 2020); however, in the literature, we see time and again, agencies are seen as practical resources by academics but not as having any useful knowledge to add. Much as women's work generally and until very recently was invisible in society and remains un(der)paid and devalued, advocates in the women's sector are often “invisible” in the generation of knowledge on violence against women.

## Critical Literature Review

In 2011, Clark and Walker re-energized the debate on victimization research with the publication of their much-cited article, “Research Ethics in Victimization Studies: Widening the Lens.” They trace the origins of ethics research from Kantian philosophy and a “deontological principlist approach,” which calls for individuals to behave morally and justly and refrain from doing harm. However, they argue that in practice, these admirable precepts which govern contemporary bioethics and the generation of knowledge that involves the participation of human subjects, have become dull and stale, following strictly rule-bound and bureaucratic procedures: “The unintended effect of the deontological approach was that it generated a system of constraints and rules that often rewards ‘rule following’ as opposed to ‘thinking ethically’” (Clark & Walker, 2011, p. 1493). In the neoliberalized, business-like, and highly competitive nature of modern universities, where research is constrained by strict deadlines as well as other funding and research ethics board requirements, they rightly critique the effect of this stale, unthinking approach to research ethics, note the temptations to “nudge method” by the researchers in the “right” direction (p. 1499)—that is, the direction that would yield publishable results in the most efficient and fastest time possible, and call for the practice of “virtue ethics,” which:

cultivates the development of ethical researchers who take a more sophisticated, moral look at the entire enterprise of a research study, from conceptualization of the question to presentation of findings in refereed journals. This does not mean ignoring IRB rules of participant protection, nor does it mean throwing out the baby with the principlist bathwater. Virtuous researchers will use a pluralistic spectrum of approaches, with the ultimate rationale of preventing exploitation of their participants. (p. 1503)

It is at this point of “pluralistic spectrum of approaches” that we call for an examination of the role of the anti-VAW sector, activists, advocates, and workers, the absence of whom in the literature on victimization research is striking. When they are mentioned, much in the manner Clark and Walker describe research ethics boards, they are featured in a perfunctory, administrative, or practical manner, as mentioned above.

Clark and Walker's argument provoked several responses from victimization scholars. Acknowledging the manifold ethical dilemmas and practical limitations which may mar victimization research, Catherine Cerulli (2011) extolled the benefits of Community-Based Research (CBR), calling for greater integration of women who had experienced abuse in the research design, much as Feminist Participatory Action Research has done decades ago, and more meaningful attempts at knowledge dissemination from research to practitioners, rather than simply relying on peer-reviewed articles. Community is the key trope in Cerulli's response to Clark and Walker, in keeping with her argument that women who are victims of intimate partner violence should be considered as a “participant class,” individual representatives of whom should be invited “at the table” throughout the stages of research, from inception and design to dissemination and implementation (Cerulli, 2011, p. 1530). Cerulli's call for inclusion is admirable, and considering the “voices” of victims of intimate partner violence would lend the significance of “lived experience” to research. It is interesting that her call for greater inclusivity of community excludes community anti-VAW organizations and practitioners. Practitioners are noted to benefit from better research dissemination; however, there is no indication that they have anything useful to offer the spectrum of research, from design to publication. Constructing the large and diverse group of “women who are victims of intimate partner violence” as a homogeneous community class, individual representatives of which may offer insight and strengthen victimization research, but completely overlooking the actual community organizations mandated to serve and support these women is significant. The invisibility of “women's work” is the conceptual framework of our argument, and it is in scholarship such as Cerulli's that this invisibility becomes even more prominent. The unquestioned, uncritiqued assumption underlying this invisibility is that community organizations in the women's sector have nothing to offer research; they may benefit from it, but they cannot contribute or strengthen it.

Mulla and Hlavka's response (2011) to the original piece by Clark and Walker argues convincingly for the inclusion of a feminist, care-based ethical approach to victimization research, in addition to, not replacing, the Kantian-based approach to ethics described by Clark and Walker above. As such, given their emphasis on feminism and care, their approach is perhaps the closest to the theoretical framework introduced in this review. It is therefore the most disappointing that in their eloquent and passionate analysis of what a feminist, care-based approach should look like, we note again the complete absence and invisibility of the actual feminist care work and woman-centered advocacy done together with victims and survivors. Mulla and Hlavka welcome these scholarly deliberations leading to “new and interesting approaches to the study of gendered violence in our society,” and use Wittgenstein's metaphor of a weaving multi-stranded rope in developing our knowledge of gender-based violence and victimization—that is, one where different approaches and perspectives may be brought together. An interdisciplinary team themselves, they recognize the value of multidimensional theoretical frameworks, as well as the importance of the social institutions which are closely involved in constructing and shaping the experiences of violence:

… we have moved away from analyses of the event of violence itself—-an epistemic object that does not easily lend itself to analysis or to generalizable findings. Instead, we find ourselves working in institutional structures that reify and validate particular experiences of suffering violence, such as the courtroom or the forensic examination. (Mulla & Hlavka, 2011, p. 1515.)

We note their emphasis on elements within the legal system—courtrooms and forensics, traditionally male-dominated spaces, which makes the absence of the female-oriented spaces of care and advocacy even more noticeable, in particular, in a piece that highlights the need for feminist ethics of care in victimization research.

Outside the corpus of responses to Clark and Walker, there is some acknowledgment of the role of community and civil society in research, in addition to the work by Sullivan et al. (2017) discussed above. Although our intention here is not to provide a comprehensive or systematic review of the literature on the relationship between academia and advocates in the anti-VAW sector, rather focusing on responding and contributing to the exchange initiated by Clark and Walker (2011), it is worth noting that some scholars have done so successfully. For example, Davidson and Bowen (2011) provide a convincing argument why academics should collaborate with community agencies when conducting research on violence against women, and how to do so. Synthesizing the literature that documents the challenges of such collaborations--for example, time investments, (lack of) trust, and power differentials--they go on to suggest practical tips on how academics should approach such agencies. The fact that these tips range from the very basic (“call the agency—ask for the Volunteer Coordinator”) to the slightly more sophisticated (listen to the service-providers—“learn about their needs and want”) indicates the nascent quality of such relationships. Indeed, the fact that Davidson and Bowen feel the need to warn prospective researchers to refrain from displaying “hierarchical and elitist” attitudes toward the staff of community agencies is quite telling. Agencies may already have sour experiences of collaboration with academics in which they felt they had been treated unkindly to the extent that they may have disillusioned the agency's view of academic research. Davidson and Bowen do not unpack the reason behind this negative history, nor do they indicate that valid and interesting knowledge on violence may be forthcoming from service providers. The relationship between academia and agencies is cast in pragmatic and mutually beneficial terms, where, as noted above, many funding opportunities are now made conditional upon such partnerships.

## Conclusion

While sitting as part of a group of “experts” on domestic violence prevention, Myrna recalled a discussion during which the chair of the group spoke about the importance of having those with “expertise.” As he continued to speak, it became clear that he meant those with credentials such as a PhD or a postsecondary degree. Also sitting at this same table were individuals with valuable expertise who did not have these credentials—those who were working within the sector, and had been for years, and those who had become advocates for domestic violence prevention after losing someone close to them. Feeling undervalued and silenced, one of those individuals later resigned from this group, despite having done significant national work in violence prevention since the death of their close family member.

In losing these voices, we lose collective knowledge. Gatekeeping and exclusionary attitudes are pervasive in academia, operating across disciplinary, racialized, and gendered lines (Franklin et al., 2021; Welsh, 2021). They are hurtful and damaging, and deepen the classed social fractures and divides which academia, ironically, should be working to overcome. In the case of victimization research and the generation of knowledge on violence against women, losing the voices of advocates and frontline service providers, or including them in a perfunctory, performative manner, weakens our collective struggle against this violence. It flies against our hard-won recognition of domestic violence as a public health issue that needs the engagement of all members of society, and it acts as a barrier in mobilizing our knowledge and putting it into action.

In a recent critical reflection, Myrna and her colleagues discuss the effects of vicarious trauma on victimization researchers, who spend their professional lives delving deeply into the minutiae accounts of devastating cruelty and brutality committed against women by their intimate partners, family members, and others (Cullen et al., 2021). Similarly, the effects of burnout and vicarious trauma on frontline workers and advocates have also been documented (Devilly et al., 2009; Moran & Asquith, 2020), although, to the point of our main argument here, the paucity of systematic literature specifically covering the women's sector and violence-against-women organizations is noticeable. A key future recommendation coming from this commentary, therefore, might be to see where such fruitful collaborations between academia and advocacy may take place in addressing vicarious and secondary trauma among practitioners and researchers, and how healing strategies may develop out of shared experiences of working and witnessing women's suffering.

We conclude these musings with insights from Hatala et al. (2016, p. 62), who call for a plurality of approaches and “plethora of voices” in studying the impact of long-term and intergenerational trauma on present-day Indigenous health inequities. They argue that such a pluralistic approach would allow space for understanding both the structural and historic damage done by colonization and oppression, as well as the individual responses and resiliency, which counteract the circulation of pathologizing, stereotypical, and simplistic narratives of “Aboriginal suffering” (p. 62). Understanding violence against women and domestic violence as a public health issue similarly rooted in longstanding structural and patriarchal inequities, we apply their insights in this sector. Indeed, women who work with survivors and victims of domestic violence have noted the intergenerational cycle of violence, as they observe people who were children when first accessing their services and support have returned as adults, either as victims or as perpetrators of violence. Making space for such voices other than those traditionally considered authoritative, understanding how different narratives operate and react in relation to each other is a key part of research into these social ills and the subsequent framings. As such, we call for a greater and more systematic inclusion and consideration of advocates and civil society in the endeavor to study and understand the complex phenomena of violence against women.
